# Data of detection and characterization of nitrated conjugated-linoleic acid (NO_2_-cLA) in LDL

**DOI:** 10.1016/j.dib.2019.105037

**Published:** 2019-12-20

**Authors:** Mauricio Mastrogiovanni, Andres Trostchansky, Homero Rubbo

**Affiliations:** Departamento de Bioquímica, Facultad de Medicina and Centro de Investigaciones Biomédicas (CEINBIO), Universidad de La República, Montevideo, Uruguay

**Keywords:** Nitro conjugated-Linoleic acid, Low-density lipoprotein, Peroxynitrite, Lipid nitration, Protein nitration

## Abstract

Under physiological and pathophysiological conditions, lipid nitration occurs generating nitro-fatty acids (NFA) with pleiotropic activities as modulation of inflammatory cell responses. Foam cell formation and atherosclerotic lesion development have been extensively related to low-density lipoprotein (LDL) oxidation. Considering our manuscript “Fatty acid nitration in human low-density lipoprotein” (https://doi.org/10.1016/j.abb.2019.108190), herein we report the oxidation versus nitration of human LDL protein and lipid fractions. Data is shown on LDL fatty acid nitration, in particular, formation and quantitation of nitro-conjugated linoleic acid (NO_2_-cLA) under mild nitration conditions. In parallel to NO_2_-cLA formation, depletion of endogenous antioxidants, protein tyrosine nitration, and carbonyl formation is observed. Overall, our data propose the formation of a potential anti-atherogenic form of LDL carrying NFA.

**Table:** Specifications Table

Subject	Biochemistry
Specific subject area	Lipid biology, oxidation, and nitration of fatty acids
Type of data	Graphs
How data were acquired	Data were obtained by i) HPLC-ESI Mass spectrometry (QTRAP4500) and analyzed with Analyst 1.6.2 software and MultiQuant (AbSciex); ii) HPLC-Fluorescent detector (Agilent 1100) and analyzed with GraphPad Prism 5.01; iii) Dot blot and densitometry analyzed with Oddysey Li-Cor software.
Data format	Raw and Analyzed
Parameters for data collection	LDL was incubated in the absence and presence of low fluxes of peroxynitrite, in phosphate buffer pH 7.4 for lipid nitration, antioxidant consumption, and protein oxidation. To determine the formation of nitrated conjugated-linoleic acid (cLA), we detected and quantified the presence of cLA in LDL. In parallel, LDL protein oxidation parameters were followed.
Description of data collection	Native and peroxynitrite oxidized-LDL were analyzed for lipid nitration, antioxidant consumption, and protein oxidation. For lipid oxidation/nitration, samples were extracted with the hexane method and analyzed. Derivatization with PTAD was performed to detect the presence of conjugated-linoleic acid in LDL, and with DNPH for protein carbonyl formation. α-Tocopherol was obtained after protein precipitation with methanol. Dot blots were performed with anti-DNPH and anti-nitrotyrosine antibodies.
Data source location	Facultad de Medicina, Universidad de la República Avda. Gral. Flores 2125, CP 11800 Montevideo, Uruguay
Data accessibility	With the article
Related article	Mastrogiovanni M., Trostchansky A., Rubbo H. Fatty acid nitration in human low-density lipoprotein. Arch Biochem Biophys. 2019 Nov 15:108190. https://doi.org/10.1016/j.abb.2019.108190.

**Table:** 

**Value of the Data** • The data show the quantification of conjugated-linoleic acid (cLA) in LDL and its nitration by peroxynitrite fluxes. • Our data presented in the article show how constant fluxes of peroxynitrite nitrate cLA in LDL in parallel to α-tocopherol depletion, apoB-100 tyrosine nitration, and protein carbonyl formation. • Studies of LC-MS/MS, HPLC-Fluorescence detection, and dot blot were done to obtain the data presented in the article, being useful tools for the detection of circulating NFA-loaded LDL in plasma samples. • Researchers studying lipid modifications and metabolism related to oxidative and inflammatory processes or chemical and analytical characterization of compounds with biological relevance may take advantage of the data set presented in this work.

## 1 Data description

**Fig. 1:**
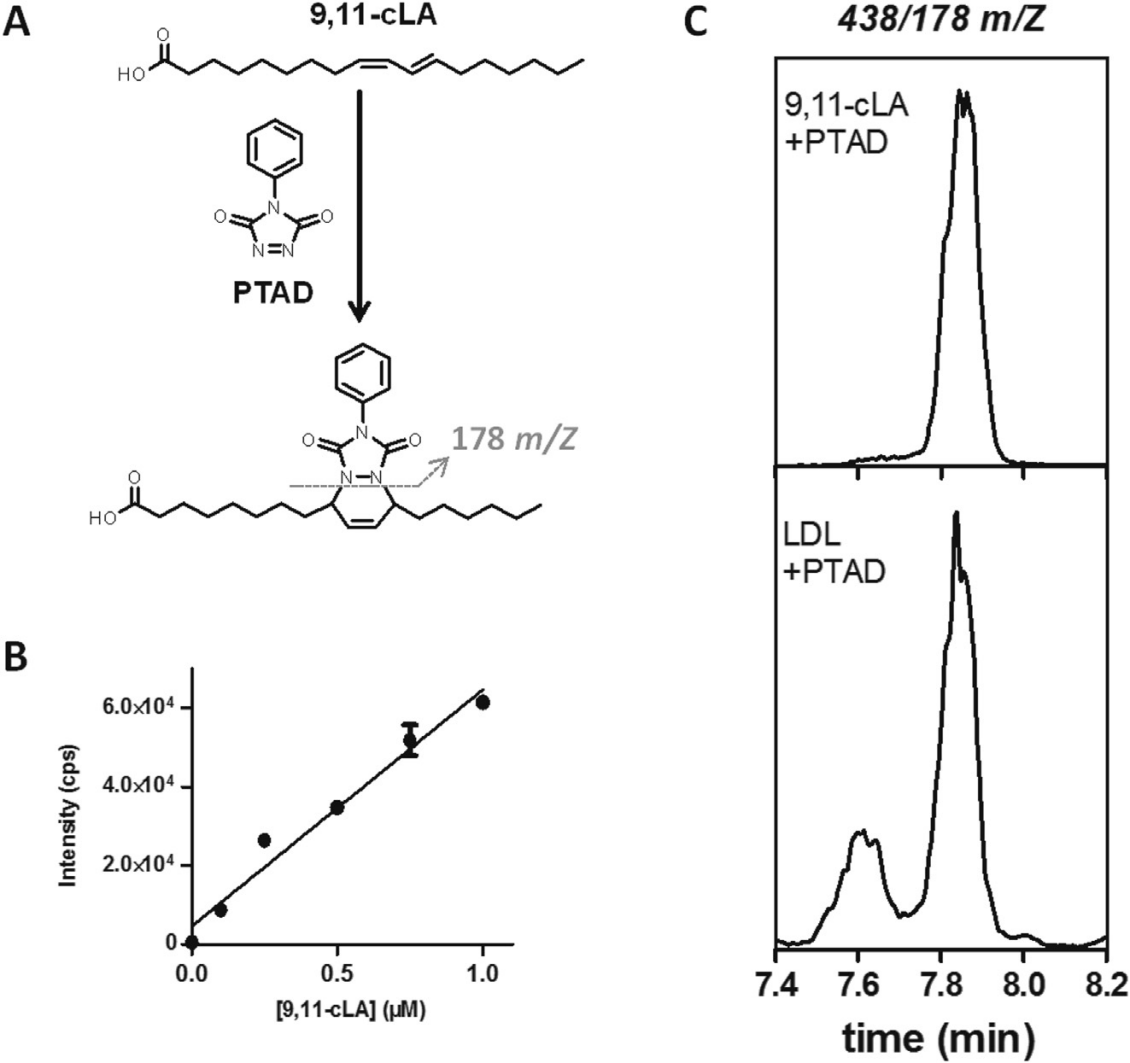
**Analysis of cLA in LDL.** Free fatty acid fractions from LDL were suspended in acetonitrile containing 10 mM PTAD that reacts with conjugated dienes forming a stable derivative that is detected and quantified by LC-MS/MS studies in the positive ion mode. *A*) Reaction scheme of PTAD addition to conjugated dienes showing the formation of the heterocyclic Diels Alder reaction product ([M+H-H_2_O]^+^, *m/z* 438 for cLA). *B*) Calibration curve obtained by LC-MS/MS using the most abundant cLA isomer, 9,11-cLA. *C*) The representative chromatogram obtained from the 9,11-cLA standard (upper panel) and the one obtained after reaction of PTAD with the free fatty acid fraction from native LDL (lower panel) following the transition *m/z* 438/178.2.

Special focus on nitration of cLA was done since previous reports found that this fatty acid is the most susceptible to nitration under physiological conditions. First, we measured cLA content in LDL samples by derivatization with PTAD on the free fatty acid fraction of LDL (Fig. 1A). Since PTAD reacts with conjugated double bonds, the 9,11-cLA standard was employed for the construction of an external calibration curve in order to quantitate cLA in LDL (Fig. 1B). The mean content of total cLA (free and esterified) on LDL samples (Fig. 1C) was 44 pmol/mg apoB100.

**Fig. 2:**
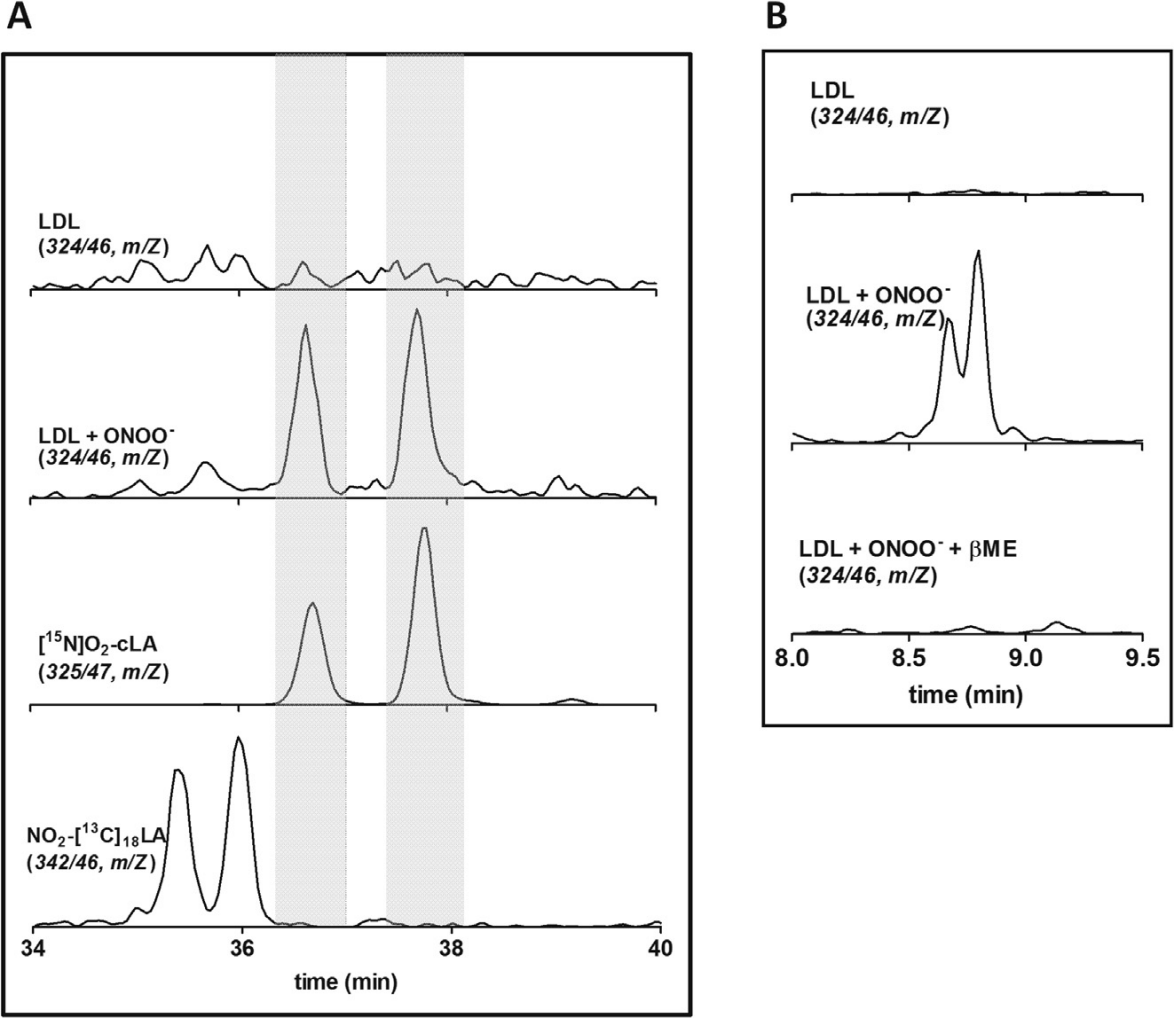
**NO_2_-cLA detection in peroxynitrite-treated LDL.***A*) LDL (3 µM) was exposed to a flux of 20 µM/min of peroxynitrite over 60 min (LDL + ONOO^−^). The presence of NFA was analyzed by LC-MS/MS. Internal standards of nitrated conjugated ([^15^N]O_2_-cLA) and non-conjugated (NO_2_-[^13^C]_18_LA) fatty acids were also analyzed and representative chromatograms are shown in lower panels. Transitions followed in each condition are indicated in the panels. *B*) The electrophilic reaction with βME was evaluated to confirm electrophilic NO_2_-cLA transitions.

Considering that cLA is present on the LDL lipidic fraction and is susceptible to nitration, we next analyzed LDL samples treated with constant low fluxes of peroxynitrite. Fig. 2 shows a representative chromatogram of HPLC-MS/MS analysis in which peaks corresponding to NO_2_-cLA were found in peroxynitrite-oxidized LDL. The HPLC method separates and identifies with high accuracy different isomers of NFA, e.g. NO_2_-LA and NO_2_-cLA. The identity of NO_2_-cLA was confirmed by comparing the retention times with the NO_2_-LA and NO_2_-cLA standards (Fig. 2A). No significant amounts of other NFA were detected (data not shown). Additional experiments were performed to confirm the presence of a nitroalkene group on the detected compounds. For this purpose, samples were incubated with BME to allow Michael addition reaction between the free thiol and the electrophilic moiety in the NFA. Afterward, samples were re-analyzed and peaks assigned to NO_2_-cLA- were completely reduced (Fig. 2B).

**Fig. 3:**
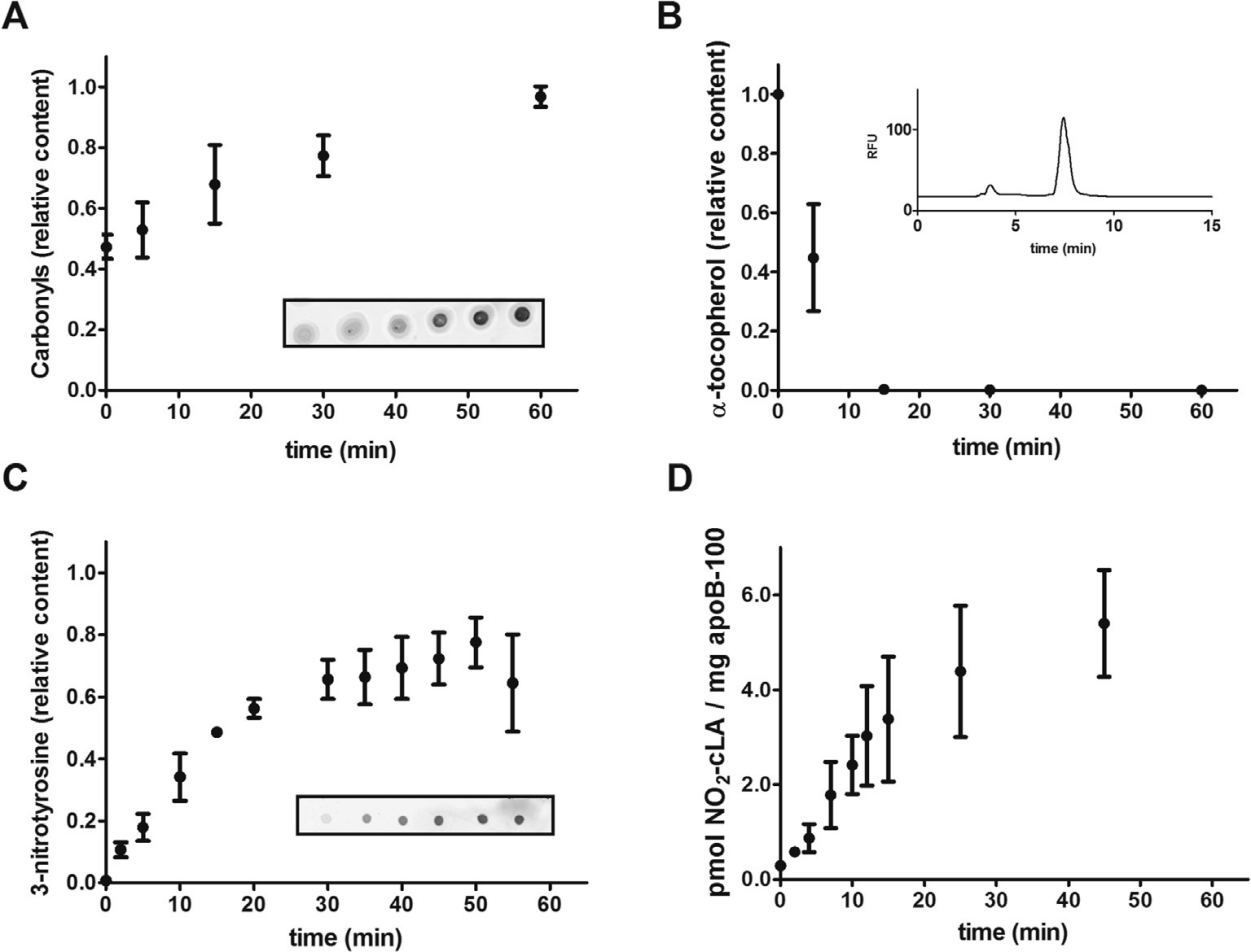
**Oxidized and nitrated LDL analysis.** Human LDL (3 µM) was exposed to a continuous flux of peroxynitrite (20 µM/min) in phosphate buffer pH 7.3. Butylated-hydroxytoluene was added at different times to stop oxidation reactions and samples were submitted to *A*) derivatization with diphenylhydrazine followed by dot-blot analysis. Representative primary data is shown on inset; *B*) MeOH extraction and reversed-phase HPLC quantitation of α-tocopherol, using a fluorescence detector (λex = 295 nm, λem = 330 nm). A representative chromatogram is shown on inset; *C*) dot-blot analysis employing polyclonal anti-3-NT antibodies. Representative primary data is shown on inset; *D*) Quantitation of NO_2_-cLA was performed by LC-MS/MS employing internal standards and a calibration curve. Data are shown from three independent experiments with n = 3. *A*, *B*, *C*, data is relative to maximum signal and represents mean ± SD. *D*, Data are related to apoB-100 content in samples and represent mean ± SD.

LDL was exposed to oxidation and the nitrating system as explained before. Dot-blot analysis of carbonyls was assessed as a fingerprint of protein oxidation. Peroxynitrite induced a hyperbolic increase in carbonyl formation in a dose-dependent manner with a saturation of carbonyl formation at 1.2 mM (Fig. 3A). As expected, α-TOH was rapidly oxidized from LDL being depleted after 10 min (Fig. 3B). All samples were submitted to the dot-blot analysis of 3-nitrotyrosine (3-NT) formation. Peroxynitrite showed a hyperbolic-like increase in 3-nitrotyrosine formation in a dose-dependent manner. Peroxynitrite infusion reaches a maximum of 3-NT formation at 2 mM (Fig. 3C).

Finally, the kinetics of LDL oxidation were compared in parallel with NFA formation. NO_2_-cLA quantitation overtime during peroxynitrite infusion was performed (Fig. 3D). The nitration curve of cLA is steepest at initial stages of incubation until it reaches a maximum at 45 min when α-TOH was totally depleted and protein oxidation almost reached a plateau. Under these experimental conditions, a mean value of 5 pmol of NO_2_-cLA/mg of apoB-100 can be found, which represents about 10% of total cLA.

## 2 Experimental design, materials, and methods

### 2.1 LDL purification

Low-density lipoprotein (LDL) was purified from healthy normolipidemic donors as previously reported [1]. The absence of other plasmatic proteins in the LDL fraction was verified by agarose gel electrophoresis and apoB-100 protein concentration was obtained by absorbance at 280 nm (ε = 1.05 (mg/mL) ^−1^. cm^−1^) [2].

### 2.2 LDL oxidation

LDL nitration was performed in 100 mM potassium phosphate buffer, pH 7.3 with 100 μM DTPA. Peroxynitrite was added for 60 min to 3 μM LDL as a continuous infusion of 20 μM/min. Due to the strong alkaline solution of peroxynitrite, the pH of all samples was checked at the end of the treatment and stayed within a 0.2 pH unit range. Reverse addition control was performed in all cases by the previous decomposition of peroxynitrite in phosphate buffer pH 7.3. After incubation with peroxynitrite, 0.025% BHT was added in order to prevent further oxidation reactions and samples were then analyzed [1,3].

### 2.3 Lipid analysis

. LDL samples (3 μM, 500 μL) were oxidized with peroxynitrite as explained above. Then, lipoprotein samples were incubated with pancreatic lipase (5 mg) and phospholipase A1 (40 U) in potassium phosphate buffer 50 mM, pH 7.4 at 37 °C under constant stirring for 1 hour. Triglyceride and phospholipid hydrolysis was followed by thin-layer chromatography [4]. After enzymatic treatment, lipids were extracted with hexane and the organic phase was separated, evaporated to dryness and resuspended in chloroform. Prior to lipid extraction, a mixture of nitrated fatty acids (NFA) internal standards -[^13^C]_18_NO_2_-LA and ^15^NO_2_-cLA- was added to each sample. A solid-phase extraction method with StrataNH2 cartridges (55 μm, 500 mg/6 mL, Phenomenex) was performed to obtain a fraction, eluted from the column with methanol, enriched in non-esterified fatty acids [[5], [6], [7], [8]].

### 2.4 Conjugated linoleic acid (cLA) detection

Dried unesterified fatty acid fractions were resuspended in an acetonitrile solution containing 10 mM of 4-phenyl-1,2,4-triazoline-3,5-dione (PTAD). This compound rapidly reacts with conjugated dienes and the resulting cLA-PTAD derivatives are detected and quantified by LC-ESI-MS/MS in the positive ion mode (*m/z* 438/178) [5].

### 2.5 Nitro-fatty acid detection

The methodology employed for NFA detection and quantitation was performed by HPLC-MS/MS using a triple quadrupole with a linear ion trap mass spectrometer (QTRAP4500, ABSciex) in the MRM mode [5,9,10].

### 2.6 β-Mercaptoethanol (BME) treatment

Dried unesterified fatty acid fractions were resuspended in 100 mM potassium phosphate buffer, pH 7.3 containing 2 mM BME, and incubated at 37 °C for 1 hr. This thiol-compound rapidly reacts with nitroalkenes forming covalent adducts with retention times and *m*/*z* different than their precursors [5,6,8,11].

### 2.7 α-Tocopherol (α-TOH) depletion

α-Tocopherol content was determined by RP-HPLC using an 1100 Agilent quaternary pump HPLC with a fluorescent detector. LDL samples were mixed with cold methanol (1:9 v:v) and centrifuged at 14000 g for 20 min; the supernatant was injected into a reverse phase C18 column (Supelco, 250 mm × 4.6 mm, particle size 5 μm) and α-TOH was eluted with MeOH and detected by fluorescence (λex = 295 nm; λem = 330 nm) [12].

### 2.8 Proteins’ carbonyls

Carbonyls protein detection and quantitation were performed by using the DNPH derivatization method. Samples were transferred to PVDF membranes and DNPH derivatization was performed in HCl 2 N. After washing and blocking the membrane, anti-DNP antibodies were used to detect carbonyls [13]. Quantitation was performed by densitometry analysis employing Oddysey Li-Cor software.

### 2.9 3-Nitrotyrosine quantitation

Nitrotyrosine detection in oxidized apoB-100 was done by dot-blot by using anti-nitrotyrosine antibodies. Quantitation was performed by densitometry analysis employing Oddysey Li-Cor software [14].
